# Design, Fabrication, and Characterization of Graphene-Silicon Nitride Integrated Mode Filters

**DOI:** 10.1021/acsphotonics.5c02651

**Published:** 2026-02-13

**Authors:** Fernando Martín-Romero, Raquel Resta, Òscar Fontelles, Miguel Sinusia Lozano, Víctor J. Gómez

**Affiliations:** Nanophotonics Technology Center, 530985 Universitat Politècnica de València, Valencia 46022, Spain

**Keywords:** graphene, waveguide, mode filter, hybrid integration, integrated photonics, 2D materials

## Abstract

We present the design, fabrication, and characterization of broadband graphene-silicon nitride integrated mode filters working in the optical C-band, centered at a wavelength of 1.55 μm. The devices presented here prevent modal crosstalk, thus avoiding signal degradation in multimode communication systems. In particular, the fabricated filters are based on a dual-mode silicon nitride waveguide, partially covered by a centered graphene nanoribbon that induces a stronger absorption for the TE_0_ mode than for the TE_1_ mode. The geometry of the design has been optimized to minimize the length and insertion losses of the device. A complete fabrication process, including the transfer and lithography of commercial graphene, has been developed. A novel approach was introduced during the etching step, which entailed the simultaneous curing of the resist to encapsulate the graphene nanoribbons prior to the deposition of the upper cladding. Finally, transmission through an array of fully fabricated filters of varying lengths was characterized with a measurement setup employing optical fiber coupling. The maximum experimentally measured contrast between the TE_0_ and TE_1_ modes is 123 dB/cm, achieved at a wavelength of 1569 nm, simultaneously with a minimum loss of −38 dB/cm for the TE_1_ mode. Overall, we fully demonstrate an integrated mode filter based on commercial graphene that paves the way for the implementation of integrated multimode optical communication systems.

## Graphical abstract

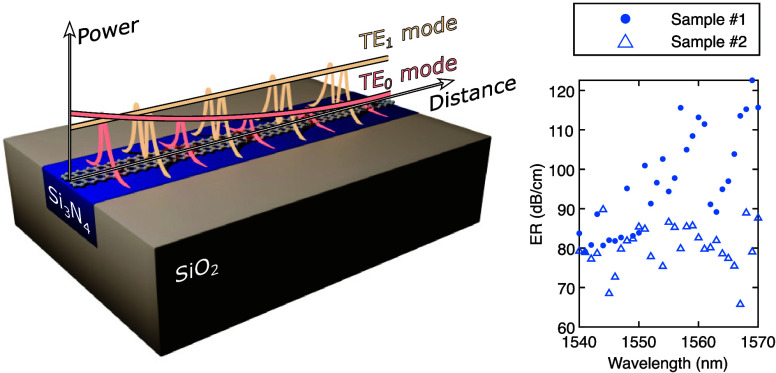

## Introduction

Hybrid graphene-silicon nitride waveguides hold a great potential to provide efficient graphene-based mode filters,^1^ photodetectors,^2,3^ modulators^4,5^ and switches.^6,7^ Graphene exhibits unique properties in a broadband wavelength range, such as ultrahigh carrier mobility, ultrafast response time and tunable Fermi energy.^8^ These properties have led to demonstrations of graphene-based photonic devices including high-speed broadband photodetectors,^9–11^ magneto-optic isolators,^12^ metasurface biosensors,^13^ all-optical switches,^14^ multimode modulators^1,15,16^ or ultrafast electro-optical modulators.^17–20^ A graphene monolayer presents an optical absorption of 2.3% at normal incidence,^21^ relatively high for 2D materials,^22^ and can be further increased by taking advantage of hybrid integration. When transferred on top of a waveguide, the in-plane evanescent field of the guided modes will couple to it, increasing its effective interaction length. The result is a larger total phase change and optical absorption.^23^ Following this concept, hybrid mode filters have been proposed where graphene acts as a selective absorber with enhanced interaction length. Graphene-polymer mode filters have been demonstrated,^24^ whereas integration on a silicon platform has only been theoretically proposed, to the best of our knowledge.^25,26^

Integrated mode filters are passive devices that selectively absorb one of several spatial modes in a waveguide. They find applications in multimode photonic integrated circuits, which are often subject to strong signal degradation due to modal crosstalk. Their main area of implementation involves optical communication systems comprising mode division multiplexing (MDM). In MDM systems, the capacity of a single wavelength channel is expanded by exploiting the propagation of higher-order guided modes.^27–30^ For the purpose of reducing mode overlap in this type of structures, various other devices have also been reported, including plasmonic subwavelength gratings,^31^ mode conversion gratings,^32^ or inverse-designed mode-routers.^33^ However, the main challenge in the realization of highly efficient multimode on-chip communication systems continues to be the suppression of modal crosstalk.

In this work, we present the design, fabrication and characterization of broadband graphene-silicon nitride integrated mode filters working in the optical C-band with potential tunability and a maximum contrast of ER = 123 dB/cm between the absorption of the TE_0_ and TE_1_ modes. The mode filters are fabricated by employing a hybrid integration strategy, which harnesses the properties of different materials in a combined integrated platform. Our straightforward approach is based on the spatial-dependent absorption generated by monolayer graphene nanoribbons on top of a low-index-contrast embedded silicon nitride (Si_3_N_4_) waveguide. Within this study, we analyze two types of passive devices. The first one corresponds to a TE_0_ filter, which aims to absorb the TE_0_ mode while transmitting the TE_1_ mode. It comprises a graphene nanoribbon centered on top of the Si_3_N_4_ core of a dual-mode waveguide (Figure 1a). The second device is a TE_1_ filter, designed for absorbing the TE_1_ mode while transmitting the TE_0_ mode. It comprises two nanoribbons along the edges of the waveguide (Figure 1b). Given the energy distribution of the transverse electric modes (TE_0_ and TE_1_), the central nanoribbon will overlap with the maximum intensity of the TE_0_ mode and the minimum intensity of the TE_1_ mode. In contrast, the outer nanoribbons will show a stronger overlap with the TE_1_ mode (Figure 1c). Therefore, the TE_0_ filter will show a higher absorption for the TE_0_ mode, and vice versa.

**Figure 1:**
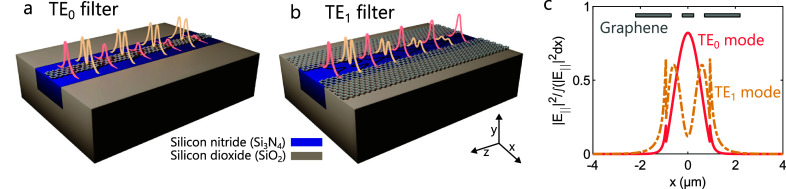
(a) Schematic view of a TE_0_ filter consisting of an embedded silicon nitride dual-mode waveguide, partially covered by a centered graphene nanoribbon. (b) Schematic view of an analogous TE_1_ filter, with graphene nanoribbons located on the sides. (c) Normalized electric field of the TE_0_ and TE_1_ modes, tangential to the graphene monolayer. The location of the graphene nanoribbons corresponding to both filters is displayed.

Given the broadband properties of graphene, the design of these filters is applicable over a wide range of optical frequencies. In the current study, we have focused on TE polarized light at a wavelength range between 1540 and 1570 nm, although simulations indicate that the TE_0_ filter would also operate effectively in the presence of TM polarized modes. The selected core material serves a dual purpose. Embedded in silicon dioxide (SiO_2_) as its upper and lower cladding, Si_3_N_4_ presents a lower index contrast than silicon. On the one hand, this causes the evanescent field on the top cladding to be more strongly coupled to graphene,^23^ increasing overall light absorption. On the other hand, the TE_1_ mode penetrates deeper to the sides and into the outer nanoribbons, improving the efficiency of the TE_1_ filter. In the following, we present the methodology and results of this work, describing how simulations were used to optimize the design, how the fabrication process was developed, and how the resulting TE_0_ filters were subsequently characterized.

## Methodology

### Simulation Workflow

The simulations were conducted with COMSOL Multiphysics, applying the finite element method (FEM) to perform a mode analysis of the filters. Two alternative methods were evaluated to simulate the optical absorption of monolayer graphene.

The first method relies on a 2D material model characterized by a surface conductivity, σ_g_. This conductivity is described by the Kubo formula.^34^ It takes into account the contribution of intraband and interband absorption processes:

$$\sigma_{\mathrm{intra}}=\frac{2k_{B}Te^{2}}{\pi \hbar^{2}}\ln\left(2\cosh\;\frac{E_{f}}{2k_{B}T}\right)\frac{i}{\omega +i\tau^{-1}}$$ 1

$$\sigma_{\mathrm{inter}}=\frac{e^{2}}{4\hbar }\left(H(\omega /2)+i\frac{4\omega }{\pi }\int_{0}^{\infty }\frac{H(\Omega )-H(\omega /2)}{\omega^{2}-4\Omega^{2}}d\Omega \right)$$ 2

$$\sigma_{g}=\sigma_{\mathrm{intra}}+\sigma_{\mathrm{inter}}$$ 3

where $H(\Omega )=\sinh\left(\frac{\hbar \Omega }{k_{B}T}\right)/\left[\cosh\left(\frac{\hbar \Omega }{k_{B}T}\right)+\cosh\left(\frac{E_{f}}{k_{B}T}\right)\right]$ , *T* is the temperature, *E*_f_ is the Fermi energy (electrochemical potential), ω = 2π*f* is the angular frequency of light, and τ is the charge relaxation time.

Even though no external doping was applied to the nanoribbons, a low Fermi energy of 0.2 eV was selected in the model to account for a potential natural doping. At a broad wavelength range around 1.55 μm, the conductivity of monolayer graphene is governed by interband transitions, since the energy of the photons is *ℏ*ω ∼ 0.8 eV > 2*E*_f_.^23^ The relaxation time is not a critical parameter in this regime, and a standard value of τ = 10^–13^ s corresponding to high-quality graphene was selected for consistency.^34^

Since a transferred graphene monolayer is about 1 nm thick, its effect on the spatial dependence of the waveguide modes can be neglected. Therefore, the electric field distribution of the TE_0_ and TE_1_ modes of the Si_3_N_4_ waveguide was computed. It can be decomposed into an in-plane and an out-of-plane component with respect to the orientation of the graphene monolayer. The in-plane component, *E*_∥_, is responsible for an induced surface current in the nanoribbons that leads to optical absorption. The propagation losses caused by graphene for each mode can be described in dB per unit length as

αp=10(log10e)Re(σg)2Pin∫wg|E∥|2 4

where *P*_in_ is the total incident power in the waveguide cross section and the square of the in-plane electric field is integrated over the width of the nanoribbon, *w*_g_.^23^

The second method is based on modeling graphene as a thin layer material with thickness *d* = 1 nm. Its permittivity was extracted from the surface conductivity of the Kubo formula as^35^

$$\varepsilon_{g}=1+\frac{i\sigma_{g}}{\omega \varepsilon_{0}d}$$ 5

Then, the TE_0_ and TE_1_ modes of the graphene-Si_3_N_4_ waveguide were simulated. The propagation losses in dB per unit length for each mode were derived from the imaginary part of the effective index, *n*_eff_:

αp=40π(log10e)Im(neff)/λ 6

This approach incorporates the residual effect of graphene on the field distribution of the modes. A comparison between both methods only revealed a relative offset of <10% in the resulting propagation losses. The first method regarding the surface current was therefore selected over the second approach, as modeling a 1 nm thick layer led to a substantially higher computational cost while providing only a small bias.

### Fabrication and Characterization Techniques

The integrated waveguides were realized on 20 mm × 20 mm samples consisting of a 300 nm LPCVD stoichiometric silicon nitride (Si_3_N_4_) core layer and a 3.26 μm SiO_2_ under cladding layer on top of a silicon substrate. They were patterned using electron beam lithography (JBX-8100FS, JEOL Ltd.) and inductively coupled plasma-reactive ion etching (ICP-RIE; STS multiplex, SPTS Technologies Ltd.) with fluorine gases (CF_4_). In- and out-coupling gratings were fabricated at both ends of the waveguides. The graphene nanoribbons on top of the silicon nitride waveguides were achieved by wet-transferring ∼1 nm thick monolayer commercial graphene (‘Monolayer Graphene Easy Transfer’, Graphenea). Details on the transfer process (Figure S2b) and the thickness of the monolayer (characterized via atomic force microscopy, AFM, Figure S3) are provided in the Supporting Information. The correct positioning of the 10 mm × 10 mm graphene sample on the patterned waveguides was attained with the support of a 3D printed PLA (polyactic acid) structure (Figure S2a). The nanoribbon geometry was defined by spin-coating the sample with hydrogen silesquioxane resist (HSQ diluted 6%; Dow Corning), and then performing electron beam lithography (JBX-8100FS, JEOL Ltd.). The development of the resist was carried out using tetramethylammonium hydroxide (TMAh; MF-319, Sigma-Aldrich). Finally, the nanoribbons were etched via oxygen plasma (30 sccm, 50 W, 60 s), and a 1 μm SiO_2_ upper cladding layer was deposited through PECVD at 200 °C with silane and nitrous oxide at a power of 270 W and a pressure of 2.7 Torr (D250L, CORIAL Plasma-Therm).

Raman spectroscopy was performed at room temperature in order to assess the presence of graphene nanoribbons on top of the Si_3_N_4_ waveguide. A confocal Raman imaging microscope (alpha 300R, WITec) was used in a backscattering setup, employing a 100× objective and a 600 g mm^–1^ grating with 2.8 cm^–1^ spectral resolution. The excitation energy (wavelength) from the laser diode module was 2.33 eV (532 nm). Measurements of the fabricated nanoribbons were performed with 5 μm × 5 μm scans consisting of 25 points per line and 25 lines per image, using an integration time of 1.5 s and a laser power of 5 mW. Single point spectra were recorded at the same laser power with two accumulations and 12 s integration time. The Raman fingerprint bands of graphene (D, G and 2D) were fitted to Lorentzian functions using the FitRaman software.^36^ The top-view geometry of the filters was inspected by means of scanning electron microscopy (SEM; JEOL IT-800SHL, JEOL Ltd.) with an acceleration voltage of 5 kV, through secondary and backscattered electrons. To characterize the cross section of the fabricated mode filters, focused ion beam (FIB) milling experiments were carried out at 30 kV and 2 nA–50 pA utilizing focused ion beam scanning electron microscopy (FIB-SEM; Zeiss AURIGA Compact). The corresponding SEM micrographs were taken at 2 kV. Compositional characterization was performed at the transverse profile by employing X-ray energy dispersive spectroscopy (XEDS; Oxford Instruments), operated at 15 keV with a working distance of 5 mm using an acceleration voltage of 5–10 keV.

The performance of the fabricated TE_0_ filters was characterized employing an experimental setup designed for on-chip transmission measurements at room temperature. It comprised a continuous wave tunable laser (OSICS ECL) with a wavelength range between 1540 and 1570 nm. The laser delivered a power output of 0 dBm through a fiber, and was subsequently routed through a 3-pad external polarization controller. It was then coupled in and out of the device under test via single-mode optical fibers oriented 10° with respect to the normal of the circuit plane. The fiber-chip alignment was performed via 3-axis stages. The power of the out-coupled light was measured using an optical power-meter (Thorlabs PM320E). Additional details on the experimental configuration, together with a schematic of the measurement setup (Figure S5) are provided in the Supporting Information.

## Results

### Design and Optimization

In order to investigate the efficiency of the mode filters, the extinction and selection ratio have been extracted from the simulated propagation losses.^25^ The propagation loss of the mode strongly absorbed by the filter is defined here as α_p;A_, while the spurious absorption of the other mode is defined as α_p;T_. The extinction ratio in dB per unit length is denoted as the difference between both parameters, ER = |α_p;A_| – |α_p;T_|. For a selected total mode contrast in dB units, *C*, it is tied to the length of the filter as *L* = *C*/ER. The selection ratio is expressed as ER divided by the propagation loss of the transmitted mode, SR = ER/|α_p;T_|. It is related to the insertion losses as |IL| = *C*/SR. As the goal is to design a device with minimum length and insertion losses, the product *L* × |IL| = *C* ^2^/(ER × SR) must be minimized. Therefore, a maximum ER × SR must be pursued.

The influence of the filter geometry on ER and SR has been studied for a central wavelength of 1.55 μm. The optimization variables corresponding to the TE_0_ filter nanoribbon are its width, *w*_g_, and its vertical offset with respect to the top of the waveguide, *h*. In the case of the two TE_1_ filter nanoribbons, the optimization variables are their width and their horizontal offset with respect to the vertical symmetry plane of the waveguide, *d*. The impact of the Si_3_N_4_ core dimensions on the extinction and selection ratio has also been explored by varying its width, *W*, and thickness, *H*.

Figure 2a,b shows the extinction and selection ratio as a function of the TE_0_ filter parameters for a fixed waveguide geometry of *W* = 1850 nm and *H* = 300 nm. These core dimensions correspond to a dual-mode waveguide. It is observed that the optimal vertical offset is 0, and the product ER × SR is maximized for a width of *w*_g_ = 500 nm. Therefore, these are the selected parameters for the fabrication of the TE_0_ filters. In the case of a TE_1_ filter with the same core geometry (Figure 2d,e), the optimal dimensions of the nanoribbons are a width of *w*_g_ = 1500 nm and a horizontal offset of *d* = 700 nm.

**Figure 2:**
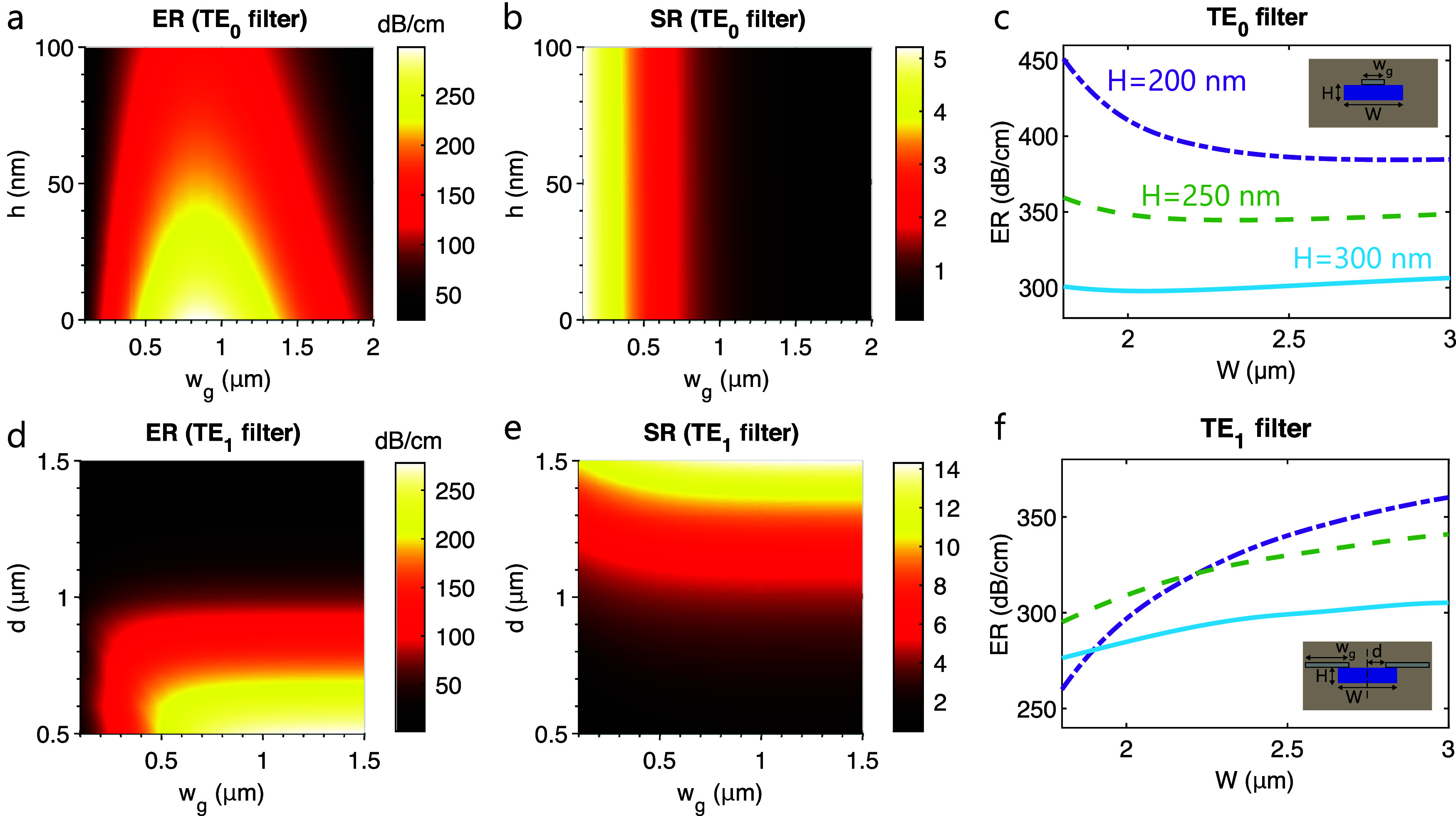
(a, b) Simulated extinction and selection ratio of the TE_0_ filter as a function of the nanoribbon width and vertical offset with respect to the waveguide core (*W* = 1850 nm, *H* = 300 nm). (c) Simulated optimal extinction ratio of the TE_0_ filter for different core dimensions. (d, e) Simulated extinction and selection ratio of the TE_1_ filter as a function of the width and horizontal offset of both nanoribbons (*W* = 1850 nm, *H* = 300 nm). (f) Simulated optimal extinction ratio of the TE_1_ filter for different core dimensions.

Regarding the influence of the waveguide core, it is worth noting that the optimal extinction ratio of both filters is generally improved for thinner waveguides (Figure 2c,f). This arises not only from the weaker confinement of the field but also from a slight redistribution of the energy of both modes within the waveguide. For TE_0_ filters with a fixed core width of *W* = 1850 nm, simulations have shown that reducing the thickness from *H* = 300 nm to *H* = 200 nm increases both the extinction and selection ratio (see Supporting Information Figure S1a,b). This is due to the increase in the evanescent field at the central nanoribbon for the TE_0_ mode and its simultaneous reduction for the TE_1_ mode (Figure S1c).

The influence of the Fermi energy or electrochemical potential of graphene on the performance of the filters has also been explored. Graphene undergoes a transition from opaque to transparent behavior when its Fermi energy is 2*E*_f_ > *ℏ*ω.^23,37,38^ Under this regime, interband absorption is no longer observed. The losses are governed by intraband transitions, which generally yield a very low absorption level. For a wavelength of 1.55 μm, graphene enters the transparent regime at *E*_f_ ≈ 0.4 eV. This is noted in the simulations, where the extinction ratio of both filters starts decreasing beyond that threshold (Figure 3a). On the one hand, this implies that a high enough natural doping of the graphene nanoribbons will negatively affect the performance of the filters. On the other hand, it suggests that an external tuning of the Fermi energy of graphene enables the operation of the device as a tunable mode filter. The tuning can be achieved, for instance, through electrical gating.^37^ With suitable electrode design, the device could potentially operate as a dual-mode modulator, representing an additional application of this platform.^1,16^

**Figure 3:**
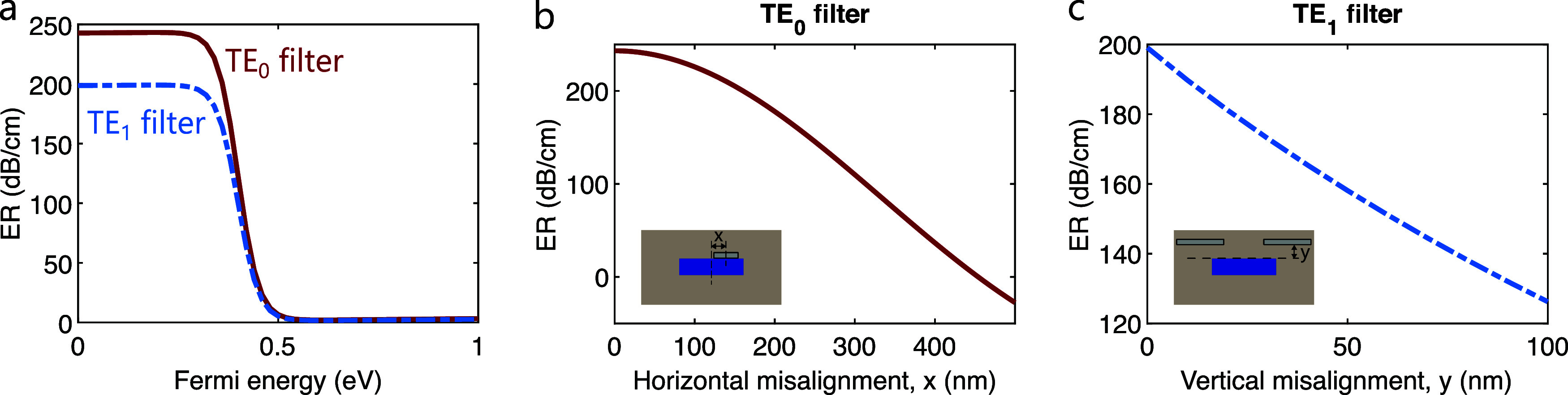
(a) Simulated extinction ratio as a function of the Fermi energy of graphene, for both filters (*W* = 1850 nm, *H* = 300 nm, *w*_g0_ = 500 nm, *h* = 0 nm, *w*_g1_ = 1500 nm, *d* = 700 nm). (b) Expected tolerance to horizontal misalignment on the position of the nanoribbon in the TE_0_ filter (*W* = 1850 nm, *H* = 300 nm, *w*_g0_ = 500 nm, *h* = 0 nm). (c) Expected tolerance to vertical misalignment on the position of the nanoribbons in the TE_1_ filter (*W* = 1850 nm, *H* = 300 nm, *w*_g1_ = 1500 nm, *d* = 700 nm).

Fabrication tolerances play an important role in the design of the mode filters. Variations in the geometrical parameters can occur during the fabrication process, potentially leading to a degradation of the device performance. For the TE_0_ filters, the potential variations arise at the nanoribbon width, *w*_g_, and its horizontal misalignment with respect to the symmetry plane of the waveguide. Inspection of the simulation results presented in Figure 2a,b reveals a large tolerance interval for ER × SR with respect to *w*_g_. It remains above 90% of its maximum value inside a width range between 300 and 600 nm. The simulated dependence of ER on the horizontal misalignment, *x*, is displayed on Figure 3b. The extinction ratio decreases below 90% for a misalignment over 120 nm. In the case of the TE_1_ filter, the main potential variation would be a vertical offset in the graphene nanoribbons due to an excessive deposit of SiO_2_ in the lateral claddings. The extinction ratio is found to be attenuated below 90% for a vertical offset of more than 30 nm (Figure 3c).

### Fabrication

The integration of graphene-silicon nitride TE_0_ filters was developed according to the optimal geometry found in the simulations. Therefore, a 500 nm wide nanoribbon was selected for a dual-mode waveguide with a width of 1850 nm and a thickness of 300 nm. Prior to the full fabrication of the mode filters, two control chips were realized. The first one consisted of four arrays of standard Si_3_N_4_ embedded waveguides, which presented low propagation losses of <5 dB/cm at a wavelength of 1.55 μm. For the second chip, a commercial 10 mm × 10 mm graphene monolayer was transferred on four analogous arrays before depositing the top cladding. The propagation losses for these graphene-covered waveguides were found to be approximately 300 dB/cm at the same wavelength.

The complete fabrication process of a mode filter is depicted in Figure 4a. Characterization of the structures was carried out at different stages through Raman scattering and SEM measurements. The first step of the process consisted on the patterning, by electron beam lithography, of the Si_3_N_4_ waveguides and grating couplers. Afterward, the commercial graphene monolayer was transferred on top of the chip using a wet-transfer method, and the chip was subsequently heated to 150 °C and stored in vacuum (<1 × 10^–3^ mbar) for 24 h. Once graphene had been transferred, HSQ resist was spin-coated onto it. The nanoribbons were patterned by electron beam lithography and the resist was developed. Then, the sample was subjected to high vacuum (<1 × 10^–6^ mbar) in order to compact the polymeric matrix of HSQ, qualifying it to be employed as a hard mask for etching the undesired graphene areas with oxygen plasma. Thus, it results in a graphene nanoribbon encapsulated by a cured HSQ layer with a refractive index (*n*_HSQ_ = 1.44) similar to SiO_2_. Finally, a 1 μm thick SiO_2_ top cladding was deposited by PECVD on top of the structure.

**Figure 4:**
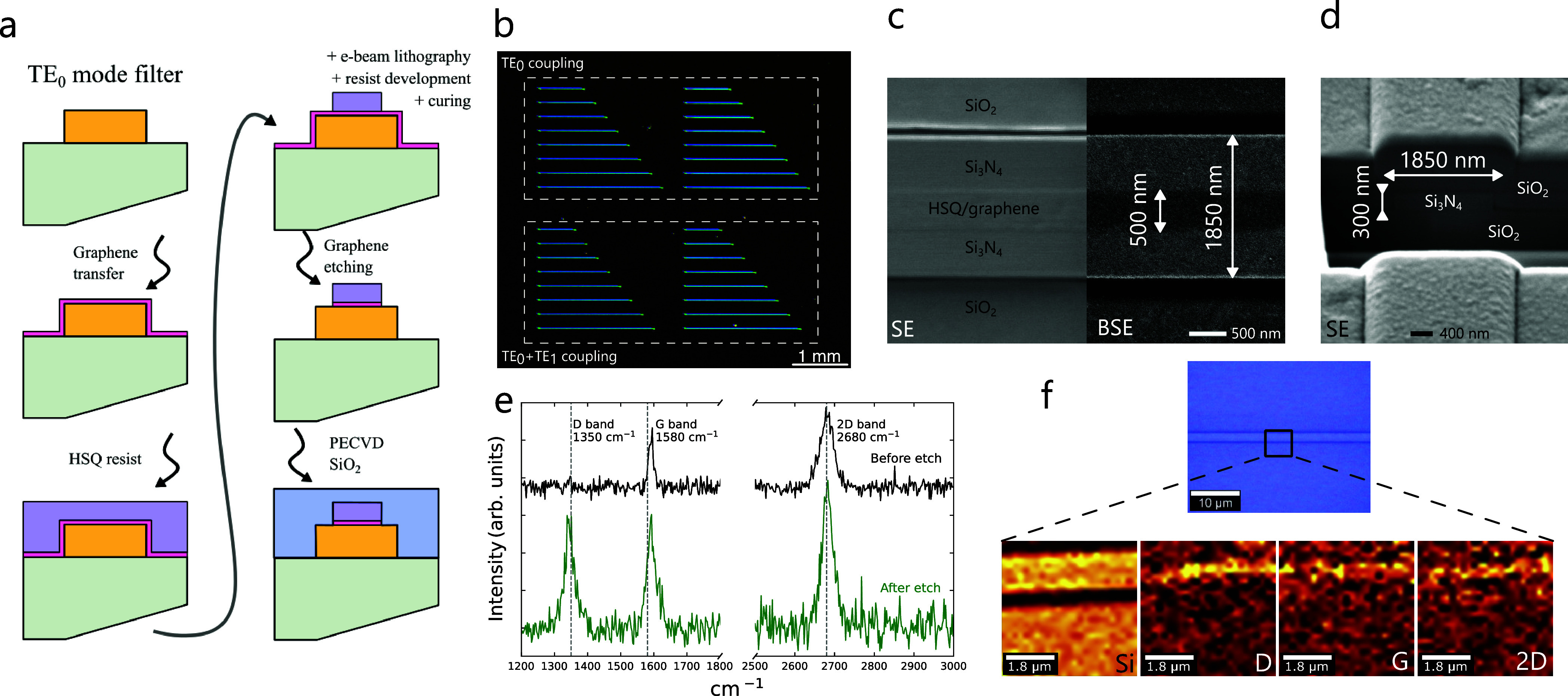
(a) Schematic of the full fabrication process for the graphene-silicon nitride TE_0_ mode filters. (b) Optical micrograph corresponding to one of the fabricated samples consisting of two pairs of mode filter arrays with TE_0_ and TE_0_–TE_1_ mixed coupling, respectively. (c) Scanning electron microscopy (SEM) images of the waveguide and nanoribbon of the TE_0_ filters, acquired using secondary electron (SE) imaging and backscattered electron (BSE) imaging. (d) SEM micrograph of the cross sections prepared by FIB milling of the TE_0_ mode filter. (e) Raman spectrum of graphene acquired before and after the fabrication of the nanoribbon. (f) Spatial dependence of the main resonances detected at a TE_0_ filter using Raman spectroscopy, corresponding to silicon (Si) and graphene (D, G, and 2D bands).

Two identical samples were designed using the developed fabrication process. An optical micrograph of one of them is shown in Figure 4b. Each of the samples contained four arrays consisting of eight mode filters, with lengths ranging from 600 to 2000 μm. The arrays were organized into two pairs, with the two arrays in each pair having the same design. The filters corresponding to the topmost pair were preceded by an input TE grating coupler, a single-mode waveguide segment and an adiabatic taper. Then, they were followed by an equivalent output grating coupler. This first configuration allowed to isolate the measurement of the optical losses of the fundamental TE_0_ mode, since the single-mode segment designed before the dual-mode filter does not support any higher-order modes. The bottom pair only featured the input and output grating couplers and the mode filter. Therefore, the losses detected using this second configuration corresponded to the incoherent simultaneous propagation of the TE_0_ and TE_1_ modes.

Figure 4c displays two electron micrographs of a TE_0_ filter before the deposition of the upper cladding, confirming that the target width was achieved for the HSQ-graphene nanoribbon and the Si_3_N_4_ core. The left portion of the figure corresponds to a secondary electron (SE) micrograph, while the right portion is a backscattered electron (BSE) micrograph. Figure 4d shows a secondary electron micrograph of the final TE_0_ filter cross section, prepared by focused ion beam (FIB) milling, verifying the correct dimensions of the Si_3_N_4_ core. Its compositional analysis, obtained by X-ray energy dispersive spectroscopy (XEDS), is described in the Supporting Information, with the corresponding spectra and concentrations displayed in Figure S4 and Table S1.

The analysis of the Raman spectroscopy measurements show how the defect related band (D band) of graphene is present (ω_D_ = 1350 cm^–1^) once the etching of graphene is carried out (Figure 4e). The resonant frequencies of the G and 2D bands allow us to identify the doping and strain of the graphene layer using the vector decomposition method.^39^ Before carrying out the etching, the resonant frequencies were ω_G_ = 1592 cm^–1^ and ω_2D_ = 2682 cm^–1^, reporting strain and doping levels of ϵ = −0.14% and *n* = 5.03 × 10^12^ cm^–2^, respectively. After etching, the resonant frequencies on the nanoribbon of the G (ω_G_ = 1591 cm^–1^) and 2D bands (ω_2D_ = 2682 cm^–1^) did not experience a significant shift resulting in strain ϵ = −0.16% and doping *n* = 3.72 × 10^12^ cm^–2^ values comparable to those obtained on the graphene layer before fabricating the graphene nanoribbons. Through this analysis, we identify the edge of the graphene nanoribbon as the main source of defects.^40^

The spatial distribution of the Raman signal corresponding to silicon (Si) and graphene is presented in Figure 4f. As it can be observed, the contribution of silicon serves to define the outline of the waveguide. Compared to it, the contributions of the resonant frequencies of graphene (D, G and 2D bands) are only present in the nanoribbon area.

### Performance Analysis

According to the chip design presented above, two types of measurements have been performed to characterize the fully fabricated TE_0_ filters. On the one hand, the transmission of the TE_0_ mode through the filters has been assessed. This parameter will be denoted as α_0_, in units of dB. On the other hand, the total transmission resulting from the incoherent propagation of the TE_0_ and TE_1_ modes through the filters has been evaluated and defined as α_mix_, in units of dB. As described, these separate cases arise from two different coupling configurations for the gratings: TE_0_ or mixed coupling. Since this study focuses on TE polarized light, the polarization of the input fiber was adjusted to align with the transverse component of the TE modes. Nevertheless, simulations indicate that the TM_0_ and TM_1_ modes supported by the waveguide would effectively pass through the filter with a low optical absorption (−56 and −10 dB/cm). This is due to their much weaker in-plane electric field relative to the plane of graphene, and it indicates that the device could also be implemented in systems supporting both polarizations.

In the TE_0_ coupling configuration, the single mode waveguide segment following the grating coupler only supports the fundamental mode. Thus, it ensures the individual propagation of the TE_0_ mode through the subsequent filter. The position of the fiber is set along the symmetry plane of the grating, where the coupling efficiency of this mode is optimized. In contrast, the mixed coupling configuration requires a more careful positioning of the fiber to allow simultaneous characterization of the TE_0_ and TE_1_ modes. No energy corresponding to the TE_1_ mode is coupled by the grating when the fiber is located along its symmetry plane. The spatial dependence of the coupling efficiency of both modes has been inspected through finite difference time domain (FDTD) simulations, performed using the FullWAVE FDTD software. Following the results, the fiber has been offset 3 μm with respect to the symmetry plane of the grating for the mixed coupling characterization. In this situation, 17% and 13% of the incident light is expected to be simultaneously coupled to the TE_0_ and TE_1_ modes, respectively. An extended explanation of the coupling configuration and fiber alignment is included in the Supporting Information, together with the simulated coupling efficiencies and a schematic of the couplers shown in Figure S6.

The normalized transmission of a single-propagating mode can be expressed as

$$T=T_{\mathrm{IL}}\times 10^{\alpha_{p}L/10}$$ 7

where *T*_IL_ is the total in- and out- coupling transmission, α_p_ is the propagation loss in dB per unit length, and *L* is the length of the filter. Hence, the total losses in dB can be written as

$$\alpha =\alpha_{\mathrm{IL}}+\alpha_{p}L$$ 8

where α_IL_ are the insertion losses in dB. By measuring the total losses as a function of the propagation length, eq 8 can be exploited to compute the propagation losses as the slope of a linear fit. This method allows to separate losses that scale with length in a device under test such as a mode filter, α_p_, from the fixed losses independent from the device length induced by any other elements (e.g., grating couplers or tapers), included in the insertion loss term, α_IL_.

In the case of two modes copropagating incoherently and coupled through fibers with lateral offset of 3 μm, the total normalized transmission is described by the equation below:

$$T_{\mathrm{mix}}=0.17^{2}\times 10^{\alpha_{p;0}L/10}+0.13^{2}\times 10^{\alpha_{p;1}L/10}$$ 9

The propagation losses in dB per unit length of the TE_0_ and TE_1_ modes are denoted as α_p;0_ and α_p;1_, respectively. The total transmission for the incoherent propagation of both modes, expressed in dB, can be written as α_mix_ = 10 log_10_(*T*_mix_). According to the simulated propagation losses, α_mix_ approaches the total loss of a single-propagating TE_1_ mode, α_1_, for sufficiently long waveguides. This behavior results from the complete filtering of the TE_0_ mode. Therefore, the approximation α_mix_ ≈ α_1_ has been applied for waveguides longer than 1 mm to determine the experimental value of α_p;1_. Finally, the validity of the approximation has been confirmed by showing that it holds for *L* > 1 mm when α_mix_ is evaluated using the experimentally obtained values of α_p;0_ and α_p;1_ at a wavelength of 1.55 μm (Figure 5a).

**Figure 5:**
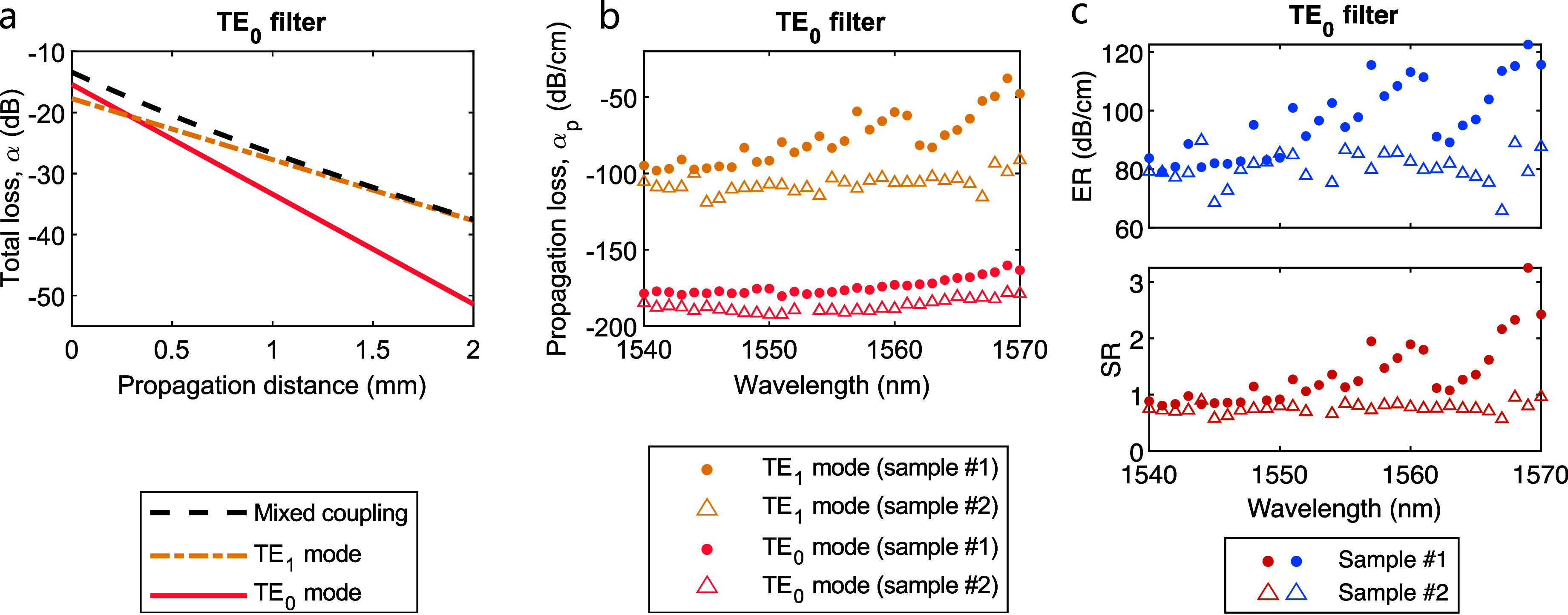
(a) Total losses in dB for a dual-mode waveguide with TE_0_, TE_1_ or mixed mode coupling, calculated at different propagation distances. (b) Propagation losses in dB/cm for the TE_0_ and TE_1_ modes, measured in two different samples of TE_0_ filter arrays. (c) Experimental extinction and selection ratio for the same two samples.

A linear fit of the TE_0_ mode total losses, α_0_, as a function of the propagation length, *L* ∈ [0.6 mm, 2 mm], has been performed for the two pairs of TE_0_-coupled filter arrays. This has enabled the evaluation of α_p;0_. At the same time, α_p;1_ has been extracted from the fitting of α_mix_ as a function of the propagation length, *L* ∈ [1 mm, 2 mm], in the two pairs of mixed-coupled filter arrays. The wavelength spectra for the propagation losses of both modes through the TE_0_ filter are presented in Figure 5b. These results demonstrate the filtering effect of the device, since the losses of the TE_0_ mode are observed to be greater than the spurious losses of the TE_1_ mode across the spectrum. Finally, applying the definition of ER and SR, both parameters have been experimentally determined (Figure 5c). The best performance has been achieved in Sample #1 at a wavelength of 1569 nm, with measured propagation losses of α_p;0_ = −160 dB/cm and α_p;1_ = −38 dB/cm, as well as an experimental extinction and selection ratio of ER = 123 dB/cm and SR = 3.25. At the central telecom wavelength of 1.55 μm, the measured average propagation losses are α_p;0_ = −184 dB/cm and α_p;1_ = −99 dB/cm, while the experimental average extinction and selection ratio are ER = 85 dB/cm and SR = 0.86. The observed variations of the propagation loss, extinction ratio and selection ratio with wavelength can be attributed to the expected redistribution of the electric field in the TE_0_ and TE_1_ modes, which modifies their overlap with the central graphene nanoribbon. This can lead to a nonflat spectral response, despite the intrinsically flat optical behavior of graphene.^25^

The propagation losses obtained from the simulations shown in Figure 2 were α_p;0_ = −327 dB/cm and α_p;1_ = −84 dB/cm at a wavelength of 1.55 μm. This corresponded to a simulated extinction and selection ratio of ER = 243 dB/cm and SR = 2.9. Therefore, the experimental results confirm the predicted operation of the TE_0_ filters (i.e., a higher absorption of the TE_0_ mode than of the TE_1_ mode in a dual-mode waveguide), although a pronounced deviation of 44% and 15% is observed respectively in the measured propagation losses of the TE_0_ and TE_1_ modes, compared to the simulations. The sources of these discrepancies could stem from the quality of graphene. The defect band present in the Raman spectrum after the fabrication process is revealed as the potential cause of the observed deviations. Other possible sources are imperfections and already discussed misalignments during the lithography of the nanoribbons. Lastly, although curing of the HSQ resist yields a material with a low refractive index similar to that of the SiO_2_ upper cladding, poor interface quality or a deviation in the refractive index could also lead to discrepancies in the results.

Table 1 provides a comparison of the demonstrated device with representative state-of-the-art devices that enable filtering of the lowest order mode. The present approach is based on a single waveguide whose length can be adjusted to trade off contrast and insertion loss according to the requirements of a given multimode photonic circuit. It allows to achieve a higher contrast at the expense of higher losses, or lower losses with moderate contrast. This design flexibility is not available in filters with a fixed geometry such as those based on subwavelength gratings,^31^ Bragg reflectors^32^ or inverse-designed structures.^33^ By relying on mode selective direct absorption rather than back-reflection or routing via small scale structural details, our device avoids potential detrimental effects in reflection-sensitive components and reduces sensitivity to fabrication imperfections. Even though some of the reported filters based on silicon on insulator (SOI) platforms achieve efficient and compact operation, they lack reconfigurability. In contrast, the hybrid integration of graphene enables active modulation or tunable filtering, providing a platform with additional key functionalities. Among graphene-based filters,^24–26^ we have experimentally demonstrated a device based on hybrid integration on a silicon nitride platform providing greater CMOS compatibility. Our operating wavelength range was limited by the tunability of the laser source used for characterization, and simulations based on similar graphene-silicon filters^26^ indicate that the expected intrinsic bandwidth of the device is substantially larger.

**Table 1:** Comparison of Key Aspects and Parameters of Our Device and Representative State-of-the-Art Devices Regarding the Selective Filtering of the Lowest Order Mode

ref	platform	2D layers	wavelength	bandwidth	propagation loss of transmitted mode	ER	insertion loss	contrast	device length	supports active modulation	result type
					(dB/cm)	(dB/cm)	(dB)	(dB)			
**this work**	graphene-Si_3_N_4_ on insulator	monolayer (commercial)	1569 nm	1540–1570 nm (limited by laser range) (ER ≳ 80 dB/cm, SR ≳ 0.9)	–38 dB/cm	123			flexible design	yes	experimental
^24^	graphene-polymer	monolayer (commercial)	1530–1620 nm	homogeneous inside range	≈ −5 dB/cm	≈20			flexible design	yes	experimental
^25^	graphene-SOI	monolayer (simulated)	1550 nm	1300–1800 nm (|IL| ≲ 2.5 dB, *C* ≳ 18 dB for target, *C* = 20 dB)	–31 dB/cm (derived from ER and SR)	310			flexible design	yes	simulation
^26^	graphene-SOI	bilayer (simulated)	1550 nm		–494 dB/cm	460			flexible design	yes	simulation
^31^	Al-SOI		1550 nm	1222–1715 nm (|IL| < 3 dB) 1433–1588 nm (*C* > 15 dB)			–0.63 dB	26.4	260 μm	no	simulation
^32^	SOI		1550 nm	1518–1570 nm (|IL| < 1 dB, *C* > 29 dB)			|IL| < 1 dB	>29	11 μm	no	experimental
^33^	SOI		1550 nm	130 nm (|IL| < 3 dB, *C* > 16.58 dB)			–1.94 dB	27.36	≈14 μm	no	experimental

## Conclusions

In this work, we have presented the design, fabrication and characterization of broadband mode filters consisting of graphene nanoribbons integrated in dual-mode silicon nitride embedded waveguides showing a maximum experimentally measured contrast of ER = 123 dB/cm between the absorption of the TE_0_ and TE_1_ modes, achieved at a wavelength of 1569 nm together with a maximum selection ratio of SR = 3.25 and a minimum loss of α_p_ = −38 dB/cm for the TE_1_ mode. The design and optimization of two distinct types of devices have been carried out. On the one hand, a TE_0_ filter that selectively absorbs the TE_0_ mode while mainly transmitting the TE_1_ mode. On the other hand, a TE_1_ filter that produces the opposite behavior. These devices show a potential tunability through the manipulation of the Fermi energy of graphene that can be achieved, for instance, via electrical gating. The geometry of the nanoribbons has been selected to minimize the length and insertion losses of the filters. With that goal, the extinction and selection ratio have been computed for varying nanoribbon and waveguide dimensions. An optimal width of 500 nm has been predicted for the nanoribbon of a TE_0_ filter with waveguide core dimensions of 1850 nm × 300 nm.

A fabrication process has been established for the TE_0_ filters. The method relies on transferring commercial monolayer graphene on top of a prepatterned silicon nitride-on-insulator circuit. The graphene is patterned by means of electron beam lithography and plasma etching, resulting in a graphene nanoribbon encapsulated by a low-index HSQ layer that protects the graphene nanoribbon from the subsequent PECVD deposition of the SiO_2_ upper cladding. Following this route, an array of TE_0_ filters of varying lengths has been fabricated. Inspection of the samples via SEM and Raman spectroscopy confirms the presence of a 500 nm wide graphene nanoribbon on each filter.

Moreover, the operation of the TE_0_ filter has been successfully demonstrated in a wavelength range between 1540 and 1570 nm. It has been confirmed that the TE_0_ mode presents higher losses than the TE_1_ mode, with an average extinction ratio of ER = 85 dB/cm at 1.55 μm. This has been achieved through fiber-coupled transmission measurements in a chip comprising grating couplers with TE_0_ or TE_0_–TE_1_ mixed coupling.

Taking into account the results discussed in this study, we present several strategies to enhance the efficiency of the mode filters. The most straightforward adjustment would entail a reduction of the Si_3_N_4_ core thickness to 200 nm, as this has been found to enhance the extinction and selection ratio of the filters. Particular attention should then be paid to the surface roughness of the Si_3_N_4_ layer before the transfer of graphene. Another option is to refine the combined etching, curing and encapsulating step in order to reduce defects in the graphene monolayer and achieve a smoother interface. Furthermore, the design of a graphene-silicon nitride ring resonator would allow an amplified mode filtering effect.^15^ These modifications are expected to greatly improve the performance of the mode filters.

The findings reported herein encompass a complete development and experimental assessment, providing a comprehensive demonstration of a hybrid integrated mode filter with low modal crosstalk that establishes a reference point for future research in the fields of two-dimensional hybrid integration and multimode on-chip optical communication systems.

## Supplementary Material



## Notes

Comparative study of filtering efficiency for a reduced core thickness; details on the graphene transfer process; AFM data for the graphene monolayer; XEDS spectra and atomic concentrations in the filter cross section; experimental setup information and schematics; extended description and schematics of the fiber-grating coupling configurations; and coupling efficiency simulations (PDF)
