# Supplementary material for: Design, Fabrication, and Characterization of Graphene-Silicon Nitride Integrated Mode Filters
Source: ACS Photonics. 2026 Feb 13;13(5):1378–87. doi: 10.1021/acsphotonics.5c02651 (PMC12965069; doi:10.1021/acsphotonics.5c02651)
Supplement: Supplementary file 1 [file ph5c02651_si_001.pdf]

Supporting Information:

Design, fabrication and characterization of graphene-silicon nitride integrated mode filters

Fernando Martín-Romero*[1], Raquel Resta[1], Òscar Fontelles[1], Miguel Sinusia Lozano[1], and Víctor J. Gómez[1]

[1]Nanophotonics Technology Center, Universitat Politècnica de València, Valencia, 46022, Spain

*Email: fmarrom@ntc.upv.es

This Supporting Information contains:

6 pages, 6 figures, 1 table

Impact of the core thickness on the filtering efficiency

The impact of decreasing the core thickness of silicon nitride from $H = 300\,\text{nm}$ to $H = 200\,\text{nm}$ has been addressed through additional mode analysis simulations in waveguides with a fixed core width of $W = 1850\,\text{nm}$. The extinction ratio (ER) and selection ratio (SR) have been obtained for a central wavelength of $1.55\,\mu\text{m}$, as a function of the optimization variables corresponding to the TE_0 filter nanoribbon (w_g and h) and the two TE_1 filter nanoribbons (w_g and d). The simulation results are presented in Fig. S1a-b, showing that a lower thickness leads to an improvement in both ER and SR. This behavior is justified by the in-plane electric field distribution of the TE_0 and TE_1 modes for $H = 300\,\text{nm}$ and $H = 200\,\text{nm}$, displayed in Fig. S1c. At the selected width, it can be observed that decreasing the core thickness enhances the evanescent field at the position of the central nanoribbon for the TE_0 mode while reducing it for the TE_1 mode.

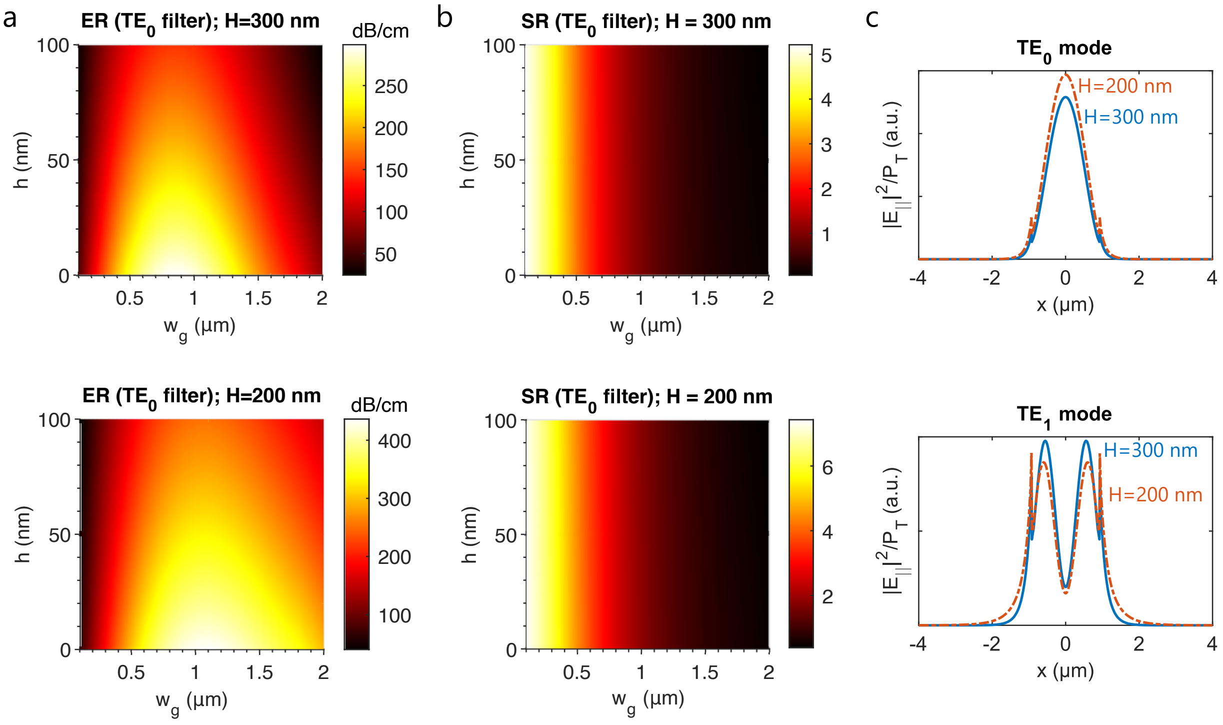

Figure S1: a. Simulated extinction ratio of a $W = 1850\,\text{nm}$ TE_0 filter for a thickness of $H = 300\,\text{nm}$ and $H = 200\,\text{nm}$. b. Simulated selection ratio of TE_0 filters with the same geometries. c. Normalized distribution of the in-plane electric field parallel to the graphene monolayer at the position of the central nanoribbon for the TE_0 and TE_1 modes, for a core width of $W = 1850\,\text{nm}$ and core thicknesses of $H = 300\,\text{nm}$ and $H = 200\,\text{nm}$.

Monolayer graphene transfer process

The wet transfer of the commercial graphene monolayer ('Monolayer Graphene Easy Transfer', manufactured by Graphenea) was carried out following the instructions provided by the supplier, with the aid of a 3D printed PLA (polyactic acid) structure. Prior to the transfer process, the patterned chip comprising the Si_3N_4 waveguides was placed inside the PLA recipient (shown in Fig. S2a). Then, the structure was filled with deionized water and the $10 \times 10\,\text{mm}^2$ commercial graphene sample was submerged in it. This sample, as described by the supplier, consisted of three layers: a top sacrificial layer, a graphene monolayer and a bottom polymer film. The first two layers floated on the water while the bottom polymer film was removed. A bottom sink present in the PLA recipient was used to control the rate of water removal. This ensures the precise positioning of the graphene on top of the chip. The resulting sample was dried in air for 30

minutes, and subsequently subject to annealing in a hot plate at 150 °C for 1 hour. Afterwards, it was stored under vacuum (below 1×10^{-3} mbar) for at least 24 hours to avoid detachment of graphene from the chip. Finally, the sacrificial layer was removed by submerging the sample in hot acetone at 50 °C for 1 hour, then in isopropyl alcohol at room temperature for another hour. The final sample was ultimately dried with N_2. A process flow schematic is depicted in Fig. S2b.

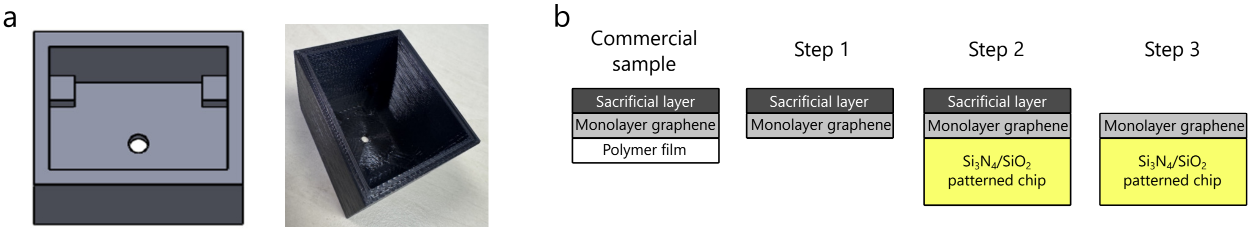

Figure S2: a. Design and photograph of the 3D printed PLA recipient utilized during the graphene wet transfer process to correctly position a graphene monolayer floating on deionized water on top of the patterned chip. b. Schematic representation of the wet transfer process as indicated by the commercial graphene supplier (Graphenea).

Atomic force microscopy on the graphene monolayer

Atomic force microscopy (AFM) measurements in tapping mode were used to verify the monolayer nature of the commercial graphene. The measurements correspond to a graphene sample transferred on a silicon substrate. Figure S3a-b shows an equalized image and a height map of the AFM topography in a region comprising the edge of the transferred graphene sample. Figure S3c displays the thickness profile along the line depicted in Fig. S3a. The peak corresponds to the edge of graphene, in which wrinkles appear due to the fabrication process, resulting in a region with higher thickness. The thickness of the monolayer is approximately 1 nm, and it has been computed as the height difference between the graphene sample and the silicon substrate, measured in the marked positions. This is the expected thickness retrieved via AFM under ambient conditions for a graphene monolayer transferred on a substrate. It must be considered that the measurement may be affected by the possible presence of physisorbed molecules on the surface between silicon and graphene and by the effects of humidity (S1).

X-ray energy dispersive spectroscopy

The material composition in a fabricated TE_0 filter cross section was analyzed utilizing X-ray energy dispersive spectroscopy (XEDS). As depicted in Fig. S4, the spectra corresponding to positions 1 and 3 were taken outside the core, in the top and bottom cladding, respectively. On the other hand, spectra 2, 4 and 5 are located inside of the Si_3N_4 core. The atomic concentrations retrieved from the spectra are displayed in Table S1, and they corroborate the expected presence of silicon and nitrogen inside of the core (corresponding to Si_3N_4) and silicon and oxide inside of the top and bottom cladding (corresponding to SiO_2).

Experimental setup for on-chip transmission measurements

Figure S5 shows a detailed schematic of the experimental setup utilized to characterize the performance of the fabricated TE_0 filters. A detailed description is provided in the manuscript (section "Methodology", subsection "Fabrication and characterization techniques"). The polarization controller was employed to ensure that the light emerging from the fiber is polarized parallel to the x-axis. A vertical camera was used to assist with the alignment between the fibers and the grating couplers. The fibers used are standard single-mode fibers (SMF) with a diameter of 9 µm. The design of the grating couplers is intended for 300 nm thick Si_3N_4 waveguides working with TE polarized light (parallel to the x-axis) incident at an angle of 10°,

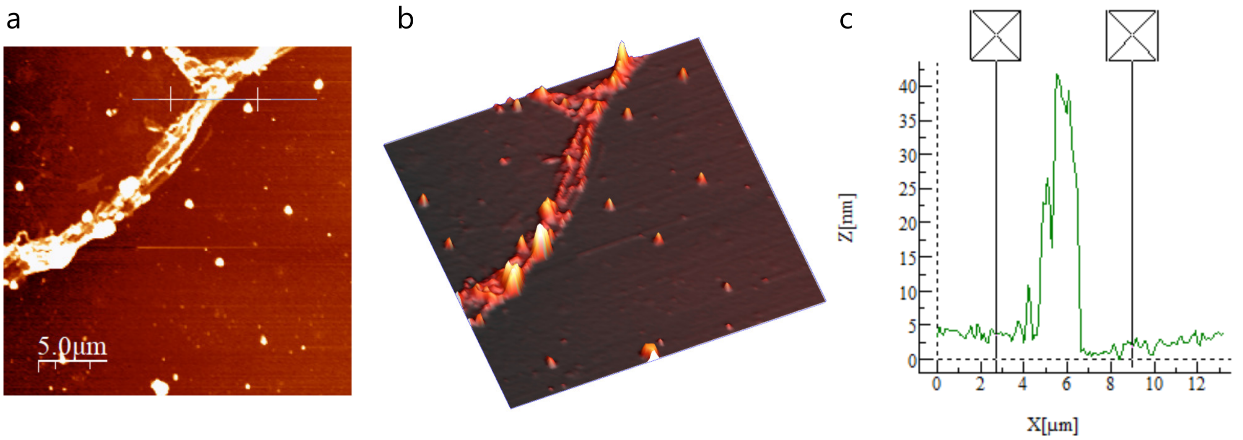

Figure S3: a. Atomic force microscopy (AFM) topography of the commercial graphene monolayer transferred on a silicon substrate (equalized image), displaying a line along which the thickness profile has been studied. b. AFM three-dimensional height map of the same sample. c. Thickness profile along the depicted line, where the left and right marked positions correspond to the height of the graphene monolayer on top of the substrate and the reference height of the silicon substrate, respectively.

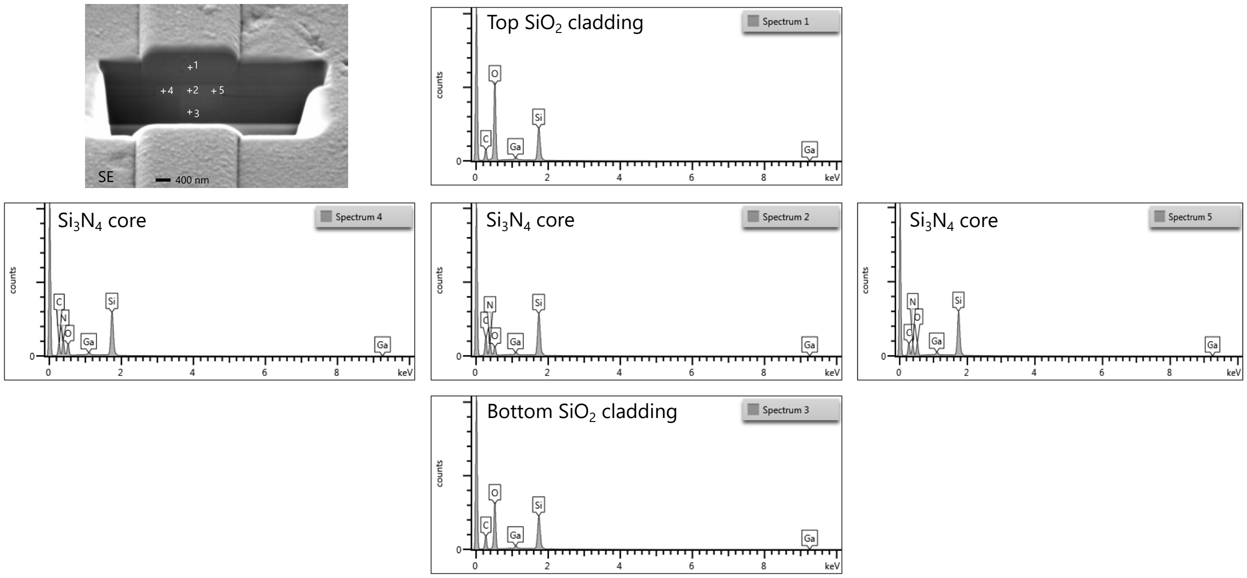

Figure S4: X-ray energy dispersive spectroscopy (XEDS) spectra acquired at the cross section of a fabricated TE_0 filter. The position at which each spectrum has been measured is indicated in a scanning electron micrograph of the cross section. Spectra 1 and 3 correspond to the top and bottom SiO_2 claddings, respectively. Spectra 4, 2 and 5 correspond to the Si_3N_4 core.

optimized for operation near the telecom wavelength of 1.55 µm. The period of the gratings is $\Lambda = 1.155\,\mu m$, while their filling factor is $FF = 0.47$.

Mode coupling

The characterization of the TE_0 filters was performed by measuring the propagation losses in two configurations: individual TE_0 coupling and mixed TE_0–TE_1 coupling. As described in the manuscript (section "Methodology", subsection "Fabrication and characterization techniques"), two distinct structures were de-

Table S1: Atomic concentration found by X-ray energy dispersive spectroscopy (XEDS) in the fabricated TE_0 filter cross section at five representative locations. SP1 and SP3 correspond to the spectra obtained inside the SiO_2 top and bottom claddings, respectively, while SP2, SP4 and SP5 correspond to spectra measured inside the Si_3N_4 core.

Element	SP1 Atomic %	SP2 Atomic %	SP3 Atomic %	SP4 Atomic %	SP5 Atomic %
C	12.85	25.74	19.23	17.29	19.5
N	0	18.51	0	22.68	16.78
O	54.15	7.86	40.18	9.76	11.2
Si	33	47.89	40.59	50.26	52.53
Total:	100	100	100	100	100

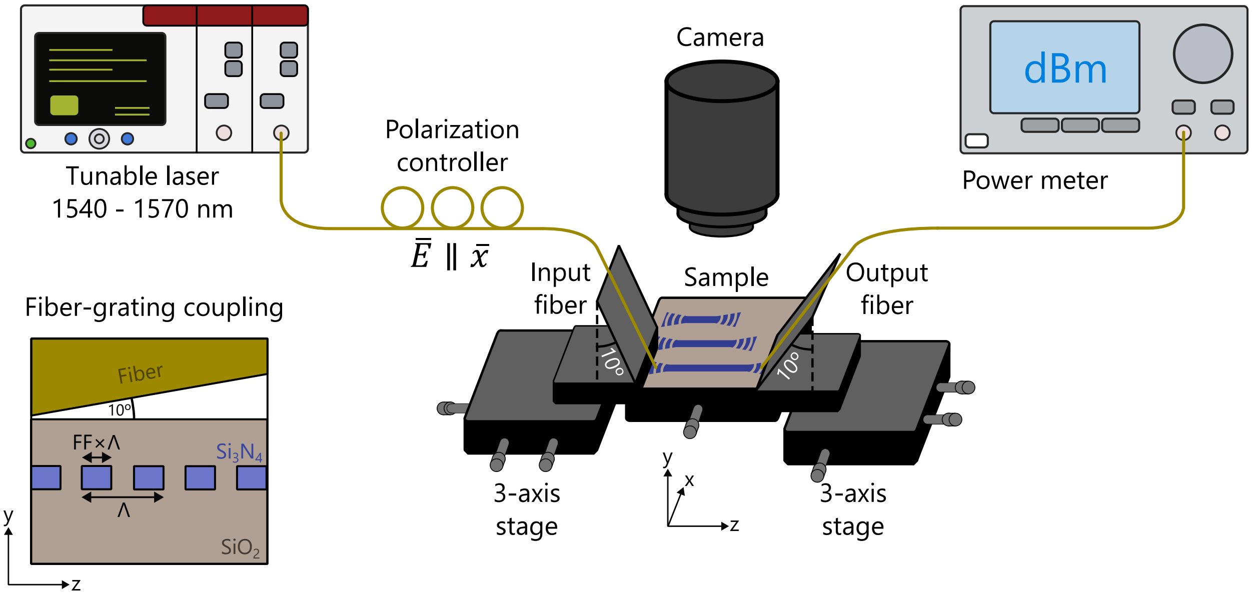

Figure S5: Schematic of the experimental setup utilized to characterize the efficiency of the fabricated TE_0 filters. It comprises a continuous wave tunable laser (OSICS ECL) with a wavelength range between 1540–1570 nm, a 3-pad external polarization controller that aligns the electric field along the x-axis, two single-mode fibers oriented 10° with respect to the normal of the circuit plane, two 3-axis stages for the alignment, and an optical power-meter (Thorlabs PM320E). The figure includes a schematic illustrating a close-up side view of the fiber coupling to one of the gratings.

signed for these measurements. The first structure consisted of the previously described grating coupler followed by a single-mode segment supporting only the TE_0 mode before the dual-mode waveguide, while the second structure comprised only the same grating followed by the dual-mode waveguide. The polarization controller in the setup was used to align the light emitted by the fiber along the x direction, parallel to the dominant E_x component of the electric field in a TE mode.

In the dual-mode waveguide, the coupling efficiency of the gratings to each mode was found to depend critically on the fiber position, as confirmed by three-dimensional FDTD simulations. The experimental setup was fully accounted for in the simulations, including the geometry and mode profile of the standard single-mode fibers, as well as their 10° tilt relative to the plane normal of the circuit. Figure S6a shows the fraction of power coupled to the TE_0 mode as a function of the x and z coordinates corresponding to the position of the center of the fiber over the plane of the circuit. Maximum TE_0 coupling occurs when the fiber is centered along the symmetry plane of the grating ($x = 0$). Figure S6b shows the coupling to the TE_1 mode as a function of the same coordinates, demonstrating that centering the fiber at the $x = 0$ plane yields zero coupling. This behavior occurs because the E_x component of the TE_1 mode is antisymmetric

with respect to $x = 0$, while both the fiber mode and the grating are symmetric. Consequently, the overlap integral of the x-polarized coupled light with the TE_1 mode must be zero.

Based on these results, measurements of the TE_0 propagation losses were conducted with the center of the fiber aligned along the symmetry plane (Fig. S6c). This configuration alone ensures individual TE_0 propagation. Any experimental residual TE_1 coupling would be further suppressed by the single-mode waveguide segment. For measurements of propagation losses under simultaneous TE_0–TE_1 coupling, the center of the fiber was displaced from the symmetry plane to enable TE_1 excitation. A displacement of 3 µm was selected, corresponding to the maximum TE_1 coupling efficiency (Fig. S6d). Fiber positioning was carefully controlled using the 3-axis stages: the symmetry plane was first identified as the x coordinate yielding maximum total transmission in the power meter, and the $x = 3$ µm plane was reached by a controlled shift of both fibers.

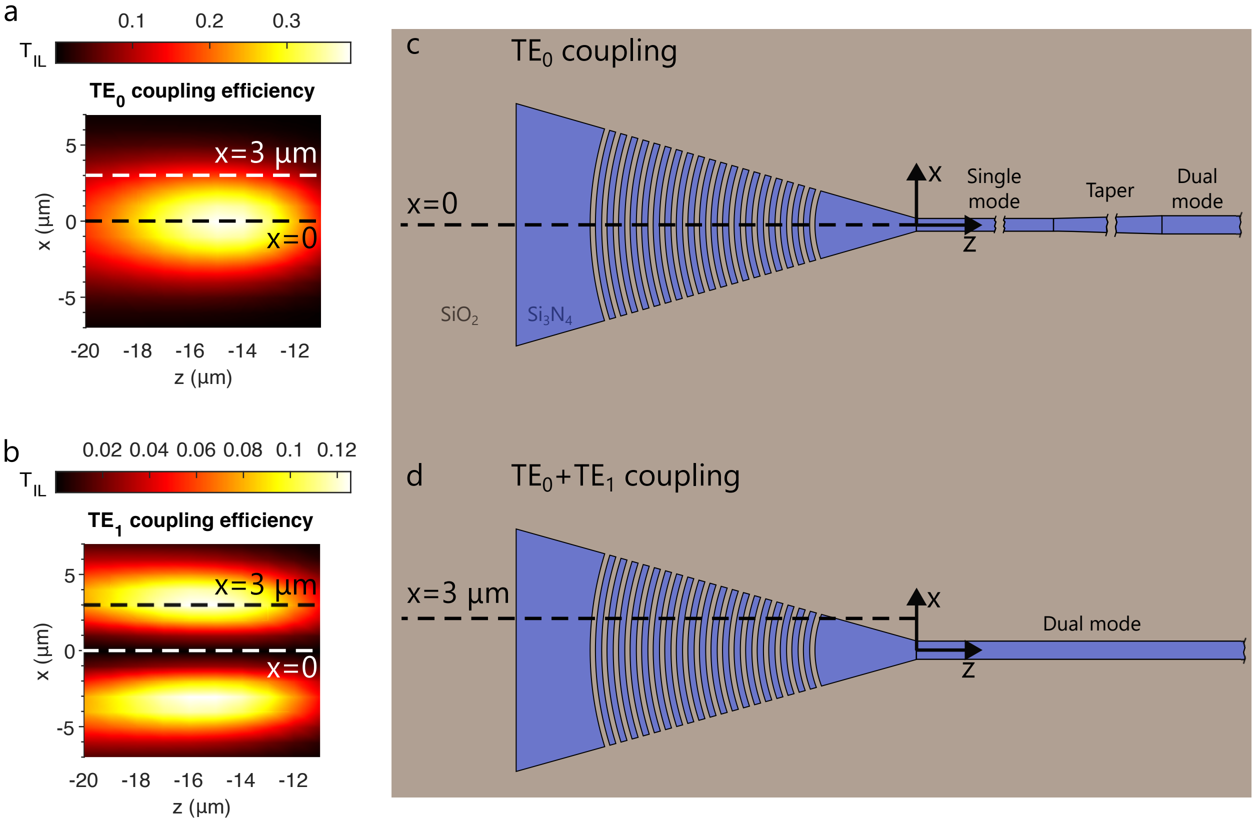

Figure S6: a. Fraction of power coupled to the TE_0 mode as a function of the x and z coordinates corresponding to the position of the fiber center over the plane of the circuit. b. Fraction of power coupled to the TE_1 mode as a function of the same coordinates. c. TE_0 coupling configuration consisting of a fiber centered along the $x = 0$ symmetry plane of the grating, a single-mode segment and a width taper connecting to the dual-mode waveguide of the filter. TE_1 coupling configuration consisting of a fiber centered along the $x = 3$ µm plane of the grating and the dual-mode waveguide of the filter.

References

(S1) Shearer, C. J.; Slattery, A. D.; Stapleton, A. J.; Shapter, J. G.; Gibson, C. T. Accurate Thickness Measurement of Graphene. *Nanotechnology* **2016**, *27*, 125704.
